# Coronary artery calcium is not universal: Population differences limit the global applicability

**DOI:** 10.21542/gcsp.2026.16

**Published:** 2026-04-30

**Authors:** Humberto Morais, Fidel Manuel Caceres-Loriga

**Affiliations:** 1 Hospital Militar Principal/Instituto Superior, Luanda, Angola; 2 Doctors HealthCare, Florida, USA

To the Editor

We read with considerable interest the recent meta-analysis by Al Hennawi et al.^1^, which synthesizes data from more than 240,000 individuals to evaluate the prognostic implications of coronary artery calcium (CAC) and its association with preventive therapies. The authors should be commended for addressing an important and timely topic. However, several key limitations warrant further discussion, particularly regarding the interpretation and global extrapolation of the findings.

A major concern is the limited geographic and ethnic representativeness of the studies included in the meta-analysis. Most contributing cohorts originate from high-income, predominantly Western populations with epidemiologic, environmental, and lifestyle profiles that differ substantially from those of low- and middle-income countries. In contrast, recent studies from sub-Saharan Africa demonstrate a strikingly low prevalence of CAC, even among adults with substantial cardiometabolic risk.

For example, Morais et al.^2^ reported notably low CAC burden in a multiethnic Angolan cohort. Their study included 211 adults (mean age 56.7 ± 9.3 years), of whom 74% were Black, 17% mixed race, and 8% Caucasian. Importantly, this cohort had a high prevalence of conventional cardiovascular risk factors - hypertension in 75%, dyslipidemia in 65%, and diabetes mellitus in 24%. Despite this significant risk burden, most participants had CAC = 0, and Black race and female sex were independently associated with lower likelihood of CAC, whereas aging, Caucasian race, diabetes, dyslipidemia, and smoking were associated with higher calcification.

Similarly, a study from Ghana found that 78.8% of adults (mean age ∼54 years) had CAC = 0, with only a very small proportion showing elevated scores. Although calcification increased with age, the large majority of participants still had no detectable CAC, underscoring the consistently low burden of calcification across multiple West and Central African populations^3^.

Likewise, the ASANTE Study from Kenya^4^ found that only ∼18% of adults ≥45 years with at least one major cardiometabolic risk factor—whether living with HIV or not—exhibited detectable CAC. These findings contrast sharply with Western cohorts of comparable age, where CAC prevalence commonly exceeds 40–60%. Importantly, prior African imaging studies have also suggested a predominance of non-calcified plaque, implying that a low CAC score does not necessarily equate to atherosclerotic “cleanliness” in these populations.

These data raise crucial questions about the external validity of the meta-analysis. If CAC prevalence and phenotype differ substantially across global regions, then the strength of association between CAC and clinical events—and the clinical usefulness of CAC as a universal decision-making tool may also vary. The meta-analysis assumes that CAC is a robust, generalizable marker across populations, yet its dataset does not adequately represent regions where the biology of calcification may follow different trajectories.

Moreover, the authors suggest that CAC can be used as a “trigger” to initiate preventive therapies such as statins or aspirin. While such an approach may be appropriate in populations where CAC strongly reflects risk, extrapolating these thresholds to regions with low calcific expression despite high cardiometabolic burden risks both under- and overtreatment. In settings where subclinical disease manifests predominantly as non-calcified plaque, a CAC-guided strategy could provide false reassurance.

For these reasons, it is essential that the conclusions of this meta-analysis be interpreted within the context of its geographic limitations. Future research should prioritize prospective, event-driven studies in under-represented populations—including Africa, Asia, and Latin America—to determine whether CAC carries equivalent prognostic weight across diverse settings.

In summary, while the meta-analysis provides valuable insights, its findings should not be regarded as universally applicable. A more nuanced, population-specific approach to CAC interpretation is warranted to ensure equitable and accurate cardiovascular risk assessment worldwide.
